# Engine Load Effects on the Energy and Exergy Performance of a Medium Cycle/Organic Rankine Cycle for Exhaust Waste Heat Recovery

**DOI:** 10.3390/e20020137

**Published:** 2018-02-21

**Authors:** Peng Liu, Gequn Shu, Hua Tian, Xuan Wang

**Affiliations:** State Key Laboratory of Engines, Tianjin University, 92 Weijin Road, Nankai District, Tianjin 300072, China

**Keywords:** Medium Cycle/Organic Rankine Cycle (MC/ORC), waste heat recovery, engine load effect, stationary CNG engine, exergy analysis

## Abstract

The Organic Rankine Cycle (ORC) has been proved a promising technique to exploit waste heat from Internal Combustion Engines (ICEs). Waste heat recovery systems have usually been designed based on engine rated working conditions, while engines often operate under part load conditions. Hence, it is quite important to analyze the off-design performance of ORC systems under different engine loads. This paper presents an off-design Medium Cycle/Organic Rankine Cycle (MC/ORC) system model by interconnecting the component models, which allows the prediction of system off-design behavior. The sliding pressure control method is applied to balance the variation of system parameters and evaporating pressure is chosen as the operational variable. The effect of operational variable and engine load on system performance is analyzed from the aspects of energy and exergy. The results show that with the drop of engine load, the MC/ORC system can always effectively recover waste heat, whereas the maximum net power output, thermal efficiency and exergy efficiency decrease linearly. Considering the contributions of components to total exergy destruction, the proportions of the gas-oil exchanger and turbine increase, while the proportions of the evaporator and condenser decrease with the drop of engine load.

## 1. Introduction

At present, increasing global concerns over climate change and energy shortages have resulted in a strong interest in energy development. In China, improving the proportion of natural gas (NG) in the primary energy mix is the main energy conservation and emission reduction strategy in the near future [1]. Natural gas, which is always used in distributed energy systems (DESs) and combined cold, heat and power (CCHP), will become the major energy suppliers for industrial parks and new towns.

Stationary compressed natural gas (CNG) engines are among of the main consumers of NG. However, most CNG engines in China are developed based on gasoline and diesel engines, hence, the thermal efficiency of most CNG engine is about 30% lower than that of diesel engines due to the restraints of engine structure [2], that is to say, a large amount of fuel energy (about 35%) is wasted through the exhaust. The conversion of exhaust waste heat into useful output is a promising approach which will improve overall thermal efficiency and save fuel. Previous researchers have shown that among the various techniques for residual energy utilization, the Organic Rankine Cycle (ORC) is a promising method for engine waste heat recovery [3,4].

Extensive studies had been conducted on waste heat recovery of internal combustion engines (ICEs) using ORC, including working fluid selection [5,6,7], cycle configuration [7,8] and economic analysis [9,10]. However, previous studies focused on the design conditions and carry out simulations based on simple thermodynamic models, whereas ORC systems fed by engine waste heat often operate far from their design point due to the changes of engine load. Previous research has shown that the available exhaust gas energy varies greatly depending on engine loads [8,11,12]. In fact, the exhaust gas flow rate and temperature variations lead the evaporator to severe off-design conditions which modify the inlet working fluid conditions until the ORC system becomes unfeasible [13]. The importance of investigating the off-design performance of ORC systems with variable engine conditions was remarked by Wang et al. [14]. The stationary ICE is widely used in distributed energy systems (DESs) and combined cold, heat and power (CCHP) systems. The stationary ICE would operate at off-design conditions when the electrical power demand decreases. According to the electricity load profiles of different buildings in a typical day [15], it is confirmed that the stationary ICE would also operate at off-design conditions for long periods of time. Regarding this, only a few works in the published literature have presented the off-design performance of ORC systems. 

Sun and Li [16] proposed a detailed off-design model of an ORC to predict system performance, and their optimization study revealed the relationship between controlled (optimal relative working fluid mass flow rate, the optimal relative condenser fan air mass flow rate) and uncontrolled variables (the heat source temperature and the ambient dry bulb temperature) on maximal net power output and thermal efficiency. Ibarra et al. [17] presented a steady-state part-load model for a small subcritical ORC system featuring a scroll expander. The part-load model can simulate the off-design conditions with the variation of four parameters: the maximum temperature of the cycle, the evaporation pressure, the condensation temperature and the expander speed. However, the attention of the authors was only focused to the off-design performance of the expander and pump without considering the off-design simulation of the vapor generator, the heat source and the condensation system. Wang et al. [18] developed an off-design static model for a solar-powered ORC system and investigated the system behavior with the change of environment temperature, thermal oil mass flow rate and solar radiation. Hu et al. [19] conducted a detailed design and off-design performance analysis based on a preliminary design of a radial turbine and exchanger based on an ORC system for a geothermal heat source, however, they identified the evaporation and condenser pressure as a control parameters and did not study any parameters affecting them. Quolin et al. [20] proposed a dynamic ORC model to optimize the working cycle conditions for a wide range of heat source and heat sink conditions. The study of Bamgbopa et al. [21] extended the findings of Quolin et al. [20] and set the evaporation temperature as a consequence of evaporator, however, the condenser was simply selected as a unit to complete the cycle at the low pressure side. In summary, the above studies mainly focused on medium-low temperature ORC systems, such as solar and geothermal powered ORC systems. 

The exhaust gas of an engine is usually at 500–900 °C which is higher than the decomposition temperature of frequently-used working fluids like R245fa and R123. If the organic working fluids are directly heated by the exhaust gas, it may cause local overheating and decomposition problems. In this case, the medium cycle (MC), an intermediate heat-transfer loop, is generally adopted to lower the exhaust temperature and ensure the safety of the working fluid [22]. The result of Li [23] showed that MC can improve the stability of ORC systems and turn all step changes into ramp changes, which makes control system more effective and robust. Gewald [22] applied an ORC with a thermal-oil cycle to recover the waste heat of several large stationary engines. By using MC, the waste heat of different engines can conveniently be recovered by an ORC system. The importance of medium cycle to transfer heat from exhaust gas to an ORC system was also noted by Vaja [24]. MC cycles can also be found in [25,26,27]. Previous studies stated that MC cycles can not only inhibit the thermal decomposition of working fluids, but also stabilize the operation of the ORC system under transient conditions. However, the off-design performance of MC/ORC under various engine loads is less studied.

The objective of this paper is to carry out an off-design performance analysis of an MC/ORC fed by the waste heat from an ICE. R245fa is selected as working fluid due to its being non-flammable and non-toxic and having relatively low environmental impact (low ODP and low GWP). The off-design system model is built by assembling the models of each component through the inlet and outlet state. The sliding pressure control is applied to balance the variation of system parameters and evaporating pressure is chosen as operational parameters. The effect of operational variables and engine load on system performance is analyzed from the aspect of energy and exergy to show its maximal working potential.

## 2. System Description

The internal combustion engine (ICE) selected in this paper is an eight-cylinder four-stroke stationary CNG engine used in a generator set. The main specification of this engine are listed in Table 1. As the engine runs in a power plant, its speed is constant (600 r/min) while its load varies under different conditions. Seven conditions of the CNG engine are picked out according to the power output range from 400 kW to 1000 kW in intervals of 100 kW. The heat balance of the engine is firstly analyzed according to data from engine tests, as listed in Table 2. The temperature of the exhaust gas is within 750–813 K and approximately 35% of the fuel energy is wasted in the exhaust gas. Therefore, it is meaningful to recover waste heat from the exhaust gas to improve the engine efficiency and reduce fuel consumption. Under the hypothesis of perfect combustion of natural gas, the composition of the exhaust gas on mass basis has been calculated at: CO_2_ = 7.11%, H_2_O = 14.22%, N_2_ = 73.4%, O_2_ = 5.27%, which is used to evaluate the gas properties.

**Figure 1:**
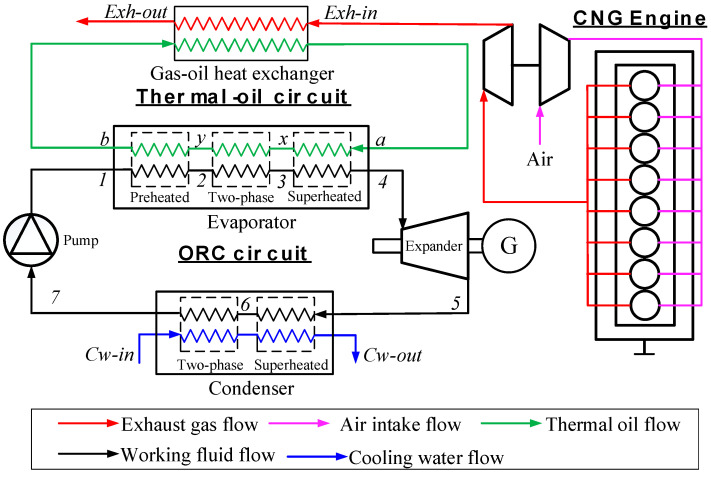
Schematic diagram of the MC-ORC system.

**Figure 2:**
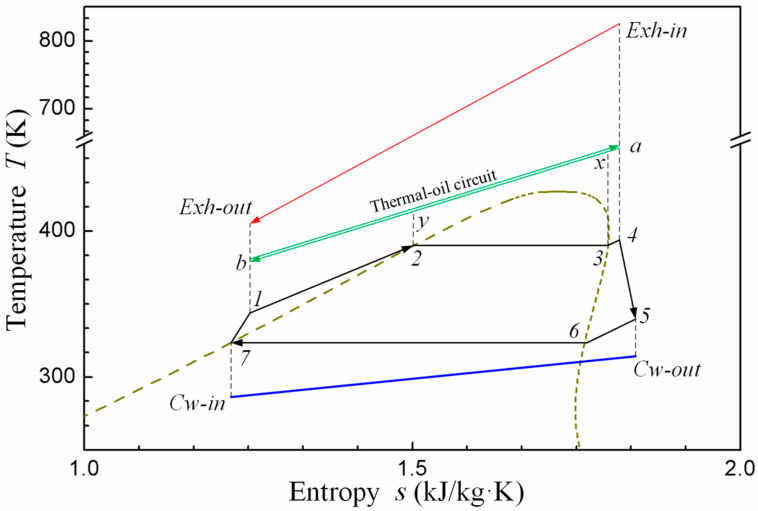
*T-s* diagram of the MC-ORC.

Figure 1 and Figure 2 show the schematic and the *T-s* diagram of the MC/ORC recovering exhaust gas waste heat from the CNG engine. As shown in figure, the red line and blue line represent the exhaust gas flow from the engine and cooling water from the water tower respectively. The black line represents the ORC system. An intermediate heat-transfer loop (the green line) is used between the exhaust gas and ORC circuit to prevent decomposition of the R245fa working fluid. The system operates as follows: the high temperature exhaust rejects heat to the thermal oil and then releases it to atmosphere. The thermal oil supplies the heat to the ORC system in the evaporator (state: a–b). At the same time, the fluid is pumped into the evaporator (state: 7–1) and becomes superheated vapor (state: 1–4). Mechanical energy is produced in the turbine during the superheated vapor expansion (state: 4–5). Finally, it is condensed (state: 5–7) and the next cycle begins. 

## 3. Mathematical Model

**Figure 3:**
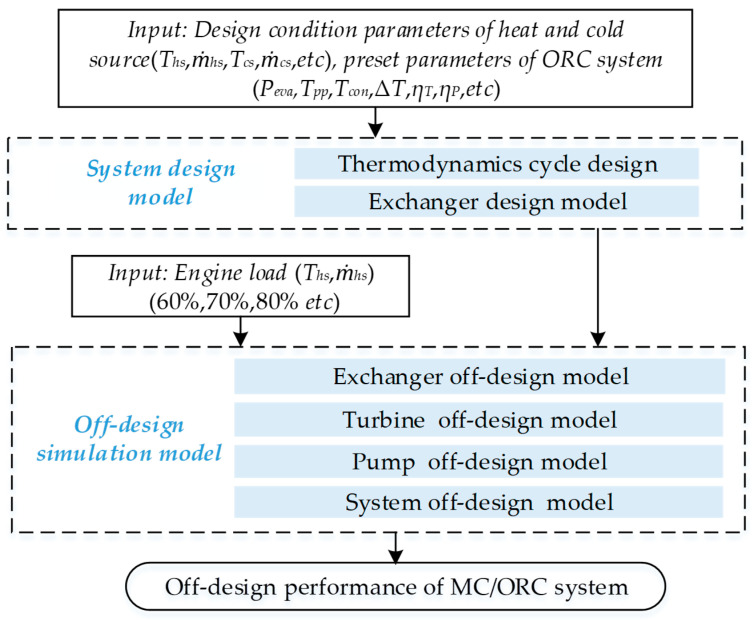
Off-design simulation modeling flow diagram.

The off-design performance simulation procedure is basically shown in Figure 3. The mathematical model in this paper includes two parts: system design model and off-design simulation model. Firstly, the ORC thermodynamic cycle is built according to the external design parameters and the main components of the ORC system are designed in the system design model. Then the above design parameters are adopted as the inputs of our off-design simulation model to analyze the system performance with variation of engine load.

### 3.1. System Design Model

#### 3.1.1. Thermodynamic Cycle Design

**Figure 4:**
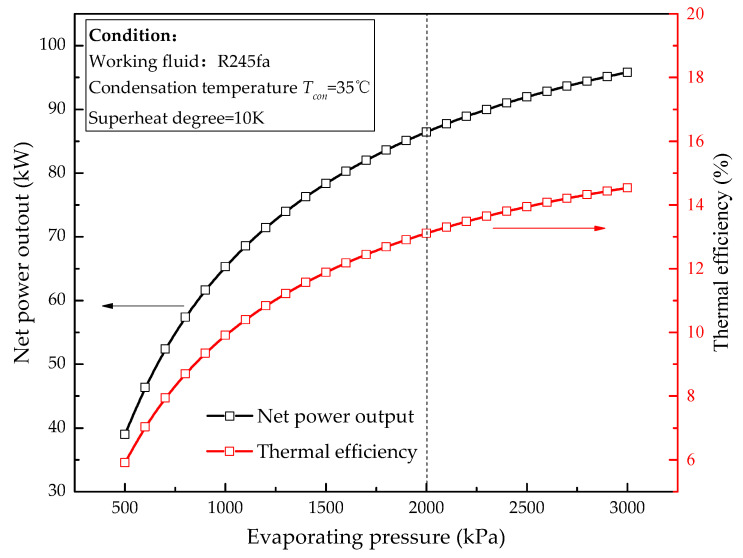
Effect of evaporating pressure on net power output and thermal efficiency.

The first step of the design procedure is to define the thermodynamic cycle under design conditions. The MC/ORC system is designed based on the engine rated conditions (100% engine load in Table 2), the thermodynamic cycle design process can be briefly described as follows: as the basis of cycle design, the condensation temperature can be determined to be 10 °C higher than the cooling water temperature. Given the condensation temperature, previous studies [5,28] presented that cycle efficiency and net power output increase first and then begin to flatten off near the critical pressure with the rise of evaporating pressure as shown in Figure 4. However, the importance of limiting evaporating pressure is remarked by many studies. Drescher et al. [29] mentioned 2000 kPa due to the safety and cost concerns. Kuo et al. [30] argued for a limit of 2500 kPa in order to keep material costs down. In this paper, 2000 kPa is set as the evaporating pressure design value. Superheat contributes negatively to the cycle efficiency and net power output for dry fluids, and is not recommended [31]. In order to avoid blade liquid corrosion, a superheating degree of 10 K is considered for design conditions. The mass flow rate of working fluid and cooling water can be determined by the Pinch Point Temperature Difference (PPTD) method. The main design parameters of MC/ORC system are summarized in Table 3.

#### 3.1.2. Heat Exchangers Design

In this section, the main heat exchanger is designed to fulfill all design specifications provided by the thermodynamic cycle at design conditions. In this paper, the TEMA E type shell-tube heat exchanger is selected due to its satisfactory pressure-bearing capacity and highly general use. The TEMA E type is the most basic types, with a single shell pass and with the inlet and outlet at the opposite ends of the shell. In gas-oil exchangers, the thermal oil flows on the tube side while exhaust gas flows on the shell side. In the evaporator, the working fluid flows on the tube side while the thermal oil flows on the shell side. As for the condenser, the working fluid flows on the shell side while the cooling water flows on the tube side. The geometric parameters of shell-tube exchangers are listed in Table 4. The heat transfer coefficient and pressure drop are determined by numerical correlations as follows: the total heat transfer coefficient based on the tube-outside surface is obtained by:

(1) $$\frac{1}{U}=\frac{1}{h_{shell}}+\frac{1}{h_{tube}}\cdot \frac{d_{o}}{d_{i}}+\frac{d_{o}}{2\lambda }\ln\left(\frac{d_{o}}{d_{i}}\right)$$

where *λ* is thermal conductivity of tube material, set as 16.3 W/mK. Then, the heat transfer area can be determined by:

(2) $$A=\frac{Q}{U\cdot \Delta T\cdot F_{t}}$$

here the Δ*T* is the log mean temperature different between the hot side and the cold side, *F_t_* is the temperature corrected factor. The limit pressure drop in heat exchangers is set as 10 kPa, which is the convergence condition for design process.

#### 3.1.3. Heat Transfer Coefficient and Pressure Drop

##### Tube side

For the cases where no phase change occurs (thermal oil in gas-oil heat exchanger, single-phase R245fa in evaporator and water in condenser), the convective heat transfer coefficient of tube-side is calculated by the Petukhov and Popov correlation [33]:

(3) $$Re=\frac{\rho \cdot v\cdot D_{eq}}{\mu }$$

(4) $$Pr=\frac{C_{p}\cdot \mu }{k}$$

(5) $$f={\left(0.782\cdot \mathrm{lg}Re-1.51\right)}^{-2}$$

(6) $$Nu=\frac{\left(f/8\right)\cdot Re\cdot Pr}{\left[12.7\cdot {\left(f/8\right)}^{0.5}\cdot \left(Pr^{2/3}-1\right)+1.07\right]}$$

(7) $$h=\frac{Nu\cdot k}{d_{i}}$$

When boiling of R245fa occurs in the evaporator, the Liu and Winterton correlation [34] is used for the convective heat transfer coefficient in two-phase zone:

(8) $$h=\sqrt{{\left(Fh_{l}\right)}^{2}+{\left(Sh_{pool}\right)}^{2}}$$

(9) $$F={\left[1+xPr_{l}\left(\frac{\rho_{l}}{\rho_{v}}-1\right)\right]}^{0.35}$$

(10) S=(1+0.055F0.1Rel0.16)−1

(11) $$h_{l}=0.023\left(\frac{\lambda_{l}}{d}\right)Re_{l}^{0.8}Pr_{l}^{0.4}$$

(12) $$h_{pool}=55p_{r}^{0.12}q^{2/3}{\left(-\mathrm{lg}p_{r}\right)}^{-0.55}M^{-0.5}$$

where *F*, *S*, *h_l_* and *h_pool_* represents the forced convective heat transfer enhancement factor, the suppression factor, liquid heat transfer coefficient and pool boiling heat transfer coefficient.

The pressure drop of tube side is given as [35]:

(13) $$\Delta P_{tube}=\Delta P_{a}+\Delta P_{N}$$

$\Delta P_{a}$ is the pressure drop along tube pass, for single phase zone:

(14) $$\Delta P_{a}=\frac{2f_{p}\cdot G^{2}\cdot L}{\rho \cdot D_{eq}}$$

*Re* < 10^5^, $f_{p}=\frac{0.316}{Re^{0.25}}$
*Re* ≥ 10^5^, $f_{p}=\frac{1}{{\left[0.79\mathrm{lg}\left(Re\right)-0.64\right]}^{2}}$

For a two phase zone:

(15) $$X=18.65{\left(\rho_{v}/\rho_{l}\right)}^{0.5}\cdot \left(1-x/x\right)\cdot \left(Re_{v}^{0.1}/Re_{l}^{0.5}\right)$$

(16) $$\phi =1+\frac{12}{X}+\frac{1}{X^{2}}$$

(17) $$\Delta P_{a,two-phase}=\phi \cdot \Delta P_{a,liquid-phase}$$

where $\Delta P_{a,liquid-phase}$ is the pressure drop in the cases of the mixture flow as liquid, calculated as above.

$\Delta P_{N}$ is the pressure drop of the inlet and outlet connecting tube, which is given by:

(18) $$\Delta P_{N}=1.5\frac{\rho v^{2}}{2}$$

##### Shell side

The geometry of the shell side is more complex than the tube side. When calculating the shell side heat transfer coefficient and pressure drop, the Delaware–Bell method is usually applied [35,36], which adopts correction factors to modify the transfer factor of an ideal tube bundle. In this paper, the baffle space is equal on the shell side, and the effect of adverse temperature gradient in laminar flow is neglected. The correction factors for the effect generated by cross flow, leak flow and bypass flow at the shell side are considered. For the single-phase fluid flowing in the shell-side, the heat transfer coefficient is calculated by:

(19) $$h_{l}=j_{H}\cdot j_{c}\cdot j_{l}\cdot j_{b}\cdot \frac{G\cdot C_{p}}{Pr^{1/3}}\cdot {\left(\frac{\mu_{bulk}}{\mu_{wall}}\right)}^{0.14}$$

where $j_{H}$, $j_{c}$, $j_{l}$, $j_{b}$ are correction factors for heat transfer factor of ideal tube bundle, baffle configuration, baffle leakage and bundle and pass partition bypass, respectively. The subscripts “bulb” and “wall” represent the working fluid properties at wall-temperature and bulk-temperature. In the condenser, the condensation heat transfer coefficient for two-phase fluid can be calculated by the correlation as follows: the heat transfer coefficient of the shell side for single phase flow can be calculated as follows [35]:

(20) $$Re_{l}=\frac{G\cdot D_{eq}\cdot \left(1-x\right)}{\mu_{l}}$$

(21) $$Pr_{l}=\frac{Cp_{l}\cdot \mu_{l}}{k_{l}}$$

(22) $$h_{l}=j_{H}Pr_{l}{}^{1/3}{\left(\frac{\mu_{l}}{\mu_{w}}\right)}^{0.14}\left(\frac{k}{D_{eq}}\right)$$

(23) $$j_{H}=0.5\left(1+B/d_{s}\right)\left(0.08Re^{0.6821}+0.7Re^{0.1772}\right)$$

(24) $$X_{tt}={\left(\frac{1-x}{x}\right)}^{0.9}\cdot {\left(\frac{\rho_{v}}{\rho_{l}}\right)}^{0.5}\cdot {\left(\frac{\mu_{l}}{\mu_{v}}\right)}^{0.1}$$

(25) $$\frac{h}{h_{l}}=1.22X_{tt}^{-0.78}$$

where subscripts “*l*” and “*v*” represent the liquid and vapor states of the fluid; *x* is the vapor quality; *B* and *d_s_* represent the baffle space and inside diameter of shell, respectively.

The pressure drop of shell side can be calculated by [35,36]:

(26) $$\Delta P_{bk}=4f_{bk}\frac{m^{2}N_{c}}{2A_{c}^{2}\rho }\left(\frac{\mu }{\mu_{wall}}\right)$$

(27) $$\Delta P_{wk}=\frac{m^{2}}{2A_{b}A_{c}\rho }\left(2+0.6N_{cw}\right)$$

(28) $$\Delta P_{\mathrm{shell}}=\left[\left(N_{b}-1\right)\cdot \Delta P_{bk}\cdot R_{b}+N_{b}\cdot \Delta P_{wk}\right]\cdot R_{l}+2\Delta P_{bk}\cdot R_{b}\cdot \left(1+\frac{N_{cw}}{N_{c}}\right)$$

### 3.2. Off-Design Simulation Model

In this section, the off-design models of the main components are built and linked together through inlet and outlet state to evaluate the off-design performance of the MC/ORC system. Before establishing these mathematical models, some assumptions are given as below: (1) the system is operating in a steady state; (2) the pressure drops in the heat exchangers and pipes are ignored; (3) the mass losses of the components are neglected in the simulation program. 

#### 3.2.1. Heat Exchanger Off-Design Model

In general, three modeling methods can be applied to predict the performance of heat exchangers: discretization model, moving boundary model and finite volumes model. When phase changes occur, the finite volumes model is inappropriate since the assumption of constant specific heat doesn’t hold. The moving boundary model treats the evaporator as a three-zone heat transfer unit, including super-heat zone, two phase zone and sub-cool zone. Then the finite volumes model can be applied in each zone. A robust iterative algorithm is required to solve the heat transfer surface area of each zone. The discretization model divides the evaporator into segments along the flow direction. In each segment, the finite volumes model can be adopted to calculate the heat and mass balance [16]. However, the discretization model is not suitable for system modeling due to higher computation cost. Therefore, the moving boundary model is utilized for the evaporator and condenser while the finite volumes model is used for the gas-oil heat exchanger.

Since there are three iteration parameters in our off-design simulation model, a triple nested iteration method is used in this paper, which has a heavy computational burden. For the convenience of calculation, the heat transfer coefficient at off-design conditions can be simply calculated by Equation (29). According to [17,37], under off-design conditions, when the mass flow rate of heat exchanger deviates from the design value, the heat transfer coefficient at off-design condition should be modified as follows:

(29) $$h=h_{des}\cdot {\left(\dot{m}/{\dot{m}}_{des}\right)}^{n}$$

where *n* depends on the heat exchanger configuration and *h_des_* is the heat transfer coefficient in the design phase. In this paper, the exponent *n* is set as 0.66 according to [37].

**Figure 5:**
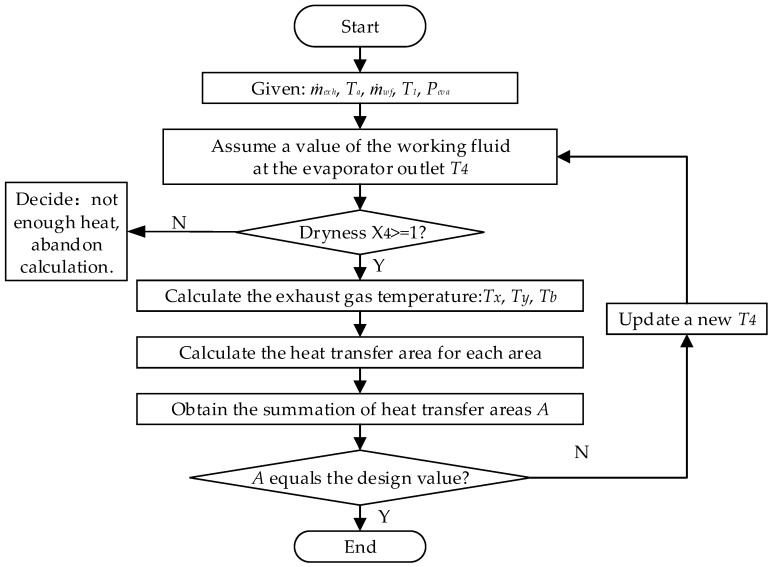
Computation process of the off-design evaporator model.

The moving boundary model treats the evaporator as a three-zone heat exchanger as shown in Figure 5.

An iterative algorithm is required to solve the heat exchanger surface area of each one. The computation procedure is shown in Figure 4. First, the outlet temperature of working fluid *T_4_* is assumed and then the thermodynamic properties of working fluid at state 1–4 are determined. Second, the exhaust gas temperatures at state *x*, *y*, *b* are obtained according to the energy conservation. Then, the heat transfer area for each zone can be calculated. If the summation of three zones area does not equal the design area, the *T_4_* is updated and a new iteration calculation is performed until the error of these two are values is less than 1%. Especially, if the vapor quality at state 4 is below 1.0, the working fluid is not completely vaporized at the outlet of the evaporator and the calculation is abandoned.

Regarding the condenser, based on the same methodology applied in [18,20], the subcooling of R245fa at the condenser outlet is assumed be constant, which can be imposed in practice by using a liquid receiver and a static pressure head between condenser and pump [20]. A similar procedure can be applied and the condenser model allows the prediction of the condensing pressure at any off-design condition.

#### 3.2.2. Turbine Off-Design Model

The turbine is the key component of the ORC system, which significantly influences the performance of the cycle. In order to evaluate the off-design performance of the turbine, a swallow capacity model based on Stodola’s equation is introduced in this paper. The rotational speed of the turbine is set as 3000 rpm to ensure the stability of the electricity frequency, since the high-speed rotational speed of a two-polar generator is generally 3000 rpm corresponding to a 50 Hz electricity frequency. Stodola’s equation, which is shown as Equation (30), determines the mass flow rate of the cycle as the function of inlet pressure, outlet pressure and the fluid density at the turbine inlet:

(30) $${\dot{m}}_{t}=K_{S}\sqrt{\rho_{in}p_{in}\left[1-{\left(\frac{p_{out}}{p_{in}}\right)}^{2}\right]}$$

where *K_s_* (*K_s_ = C_d_ ·A*) is Stodola’s coefficient, which is the product between the coefficient discharge and the equivalent nozzle cross area at the inlet. In this expression, the *K_s_* is a constant that is fitted at the design condition. Therefore, the output power can be calculated as:

(31) $${\dot{W}}_{t}={\dot{m}}_{t}(h_{in}-h_{out})$$

(32) $$h_{out}=h_{in}-\eta_{t,is}(h_{in}-h_{out.is})$$

where $h_{out.is}$ is the enthalpy at the exit state of the isentropic process. The isentropic efficiency of the turbine is calculated starting from the isentropic efficiency at design conditions (0.8) and multiplying it by two correction factors. One correction factor (*CF_1_*) is related to the variation of *u*/*c*_0_ that results from the variation of enthalpy drop under off-design conditions The term. *u* is the impeller tangential speed and *c*_0_ is the spouting velocity ($c_{0}=\sqrt{2\cdot H_{is}}$). The other factor (*CF_2_*) is related to the variation of the mass flow rate. The curve of *CF_1_* and *CF_2_* is obtained from [24]. This model has been widely used by several authors [37,38,39] to predict the off-design performance of turbines. The main characteristics of the expander are listed in Table 5.

(33) $$\eta_{t,is}=CF_{1}\cdot CF_{2}\cdot \eta_{t,is,des}$$

(34) $$CF_{1}=a_{1}{\left(u/c_{0}\right)}^{3}+b_{1}{\left(u/c_{0}\right)}^{2}+c_{1}(u/c_{0})+d_{1}$$

(35) $$CF_{2}=a_{2}{\left(\dot{m}/{\dot{m}}_{des}\right)}^{3}+b_{2}{\left({\dot{m}/\dot{m}}_{des}\right)}^{2}+c_{2}({\dot{m}/\dot{m}}_{des})+d_{2}$$

#### 3.2.3. Pump Off-Design Model

The efficiency of the pump at off-design conditions is determined by a third-degree polynomial of the ratio of the inlet volumetric flow with respect to the design point [40]. The coefficients of the polynomial are fitted according to the performance curve of a commercial pump [38]. The pump maximum efficiency is set equal to 0.8 and it is assumed to occur at the design conditions. The main characteristics of the pump are listed in Table 5. The power consumption related to the pump can be calculated as well:

(36) $$\eta_{pp,is}/\eta_{pp,is,des}=a{\left(\dot{V}/{\dot{V}}_{des}\right)}^{3}+b{\left(\dot{V}/{\dot{V}}_{des}\right)}^{2}+c\left(\dot{V}/{\dot{V}}_{des}\right)+d$$

(37) $$h_{out}=h_{in}+(h_{in}-h_{out.is})/\eta_{pp,is}$$

(38) $${\dot{W}}_{pp}=\dot{m}(h_{in}-h_{out})$$

The pump has little influence on the system performance and this methodology is acceptable to predict its performance.

#### 3.2.4. System Integration

**Figure 6:**
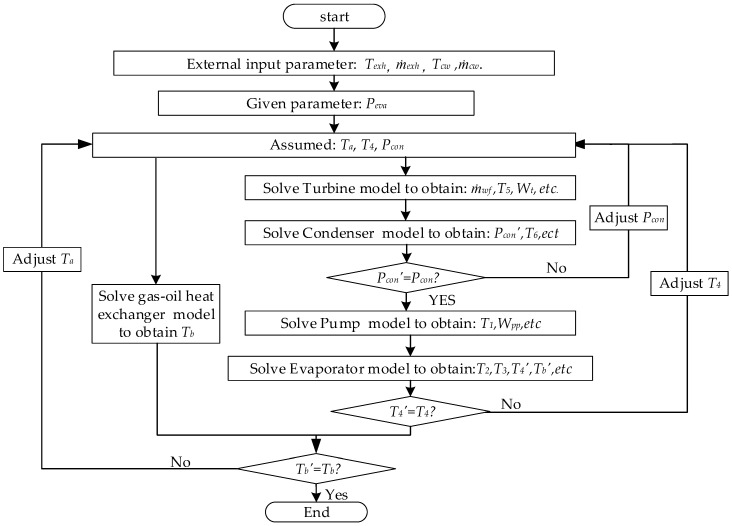
Computation process of the off-design simulation of MC/ORC model.

The off-design system model is built by interconnecting the off-design component models together through the inlet and outlet state, which can reflect the real physic connections that occur between the components of the power plant. When the operational conditions deviate from the design point, the sliding pressure control is applied to balance the variation of system parameters and evaporating pressure *P_eva_* is chosen as operational variable. The external inputs of the numerical ORC model include the exhaust gas temperature *T_exh-in_*, exhaust gas flow rate *ṁ_exh_*, cooling water flow rate *ṁ_cw_*, cooling water temperature *T_cw-in_*. The flow chart of the simulation procedure is shown as Figure 6.

To evaluate the performance of the system, the proposed criteria are as follows: firstly, the concept of waste heat utilization rate (UR) is presented to clarify the heat recovery capability of MC/ORC system. The waste heat utilization rate is the ratio of heat recovered from the certain source to the maximum heat available in this heat source. It should be noted that the maximum available heat from exhaust gas is the heat rejected when the exhaust gas is cooled to the acid dew point:

(39) $$U_{g}=\frac{{\dot{m}}_{g}\cdot C_{p,}{}_{g}\cdot \left(T_{g,in}-T_{g,out}\right)}{{\dot{m}}_{g}\cdot C_{p,}{}_{g}\cdot \left(T_{g,in}-T_{g,dew}\right)}$$

Then, the net power output and thermal efficiency are given:

(40) $${\dot{W}}_{net}={\dot{W}}_{t}-{\dot{W}}_{pp}$$

(41) $$\eta_{th}=\frac{{\dot{W}}_{net}}{Q_{in}}=\frac{{\dot{W}}_{net}}{c_{exh}\cdot {\dot{m}}_{exh}\cdot \left(T_{exh-in}-T_{exh-out}\right)}$$

Exergy analysis is carried out based on the second law of thermodynamics considering the irreversibility of the system. The exergy value at any state *i* can be defined as:

(42) $${\dot{E}}_{i}=\dot{m}\left[\left(h_{i}-h_{0}\right)-T_{0}\left(s_{i}-s_{0}\right)\right]$$

where the subscript “0” refers to the ambient condition, which is set as 298.15 K and 101.3 kPa. The irreversibility of each component can be expressed as:

(43) $${\dot{I}}_{i}=\sum {\dot{E}}_{in}-\sum {\dot{E}}_{out}-{\dot{W}}_{i}$$

Hence, the exergy efficiency can be calculated as:

(44) $$\eta_{ex}=\frac{{\dot{W}}_{net}}{{\dot{E}}_{exh-in}-{\dot{E}}_{exh-out}}$$

## 4. Results and Discussion

In this paper, the MC/ORC system is designed based on the rated engine condition (100% engine load) of the CNG engine. The sliding pressure control method is adopted since it has been proven to provide high efficiency in part-load operations [18]. Sliding pressure control means that the evaporating pressure is the main control variable to meet the steady operation of turbine, and the regulation valve and stop valve at the turbine inlet are both fully opened to eliminate the throttling loss. The evaporating pressure is chosen as the operational variable. The effect of the operational variable and engine load on system performance is analyzed and the best operation scenarios are defined to show the maximal working potential. To ensure safe operation, the feasible operation points are subject to the following constraints: (a) the working fluid is completely vaporized at the outlet of evaporator (superheat degree Δ*T* ≥ 0) to avoid blade liquid corrosion [41]. (b) the exhaust gas temperature at the evaporator outlet should be higher than the acid dew temperature to avoid exhaust gas acid corrosion.

### 4.1. Evaporating Pressure Effect on MC/ORC System Performance

**Figure 7:**
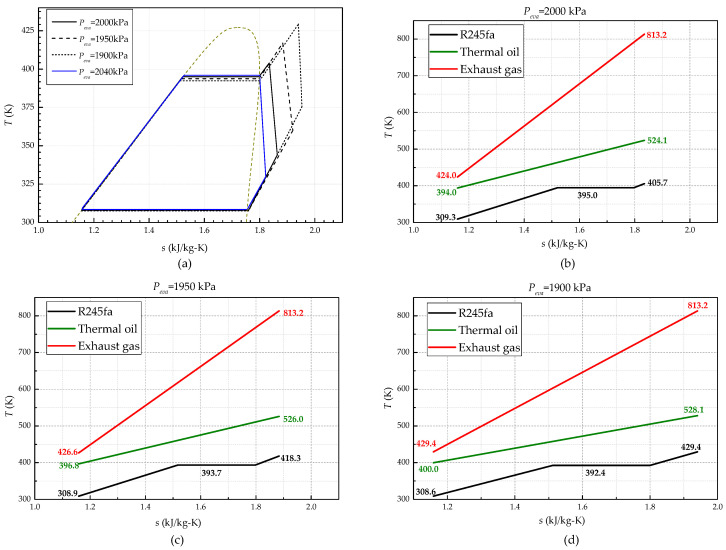
Variation of the thermodynamic cycle with *P_eva_* under 100% engine load: (**a**) Thermodynamic cycle variation; (**b**) Heat transfer profiles at *P_eva_* = 2000 kPa; (**c**) Heat transfer profiles at *P_eva_*=1950 kPa; (**d**) Heat transfer profiles at *P_eva_* = 1900 kPa.

Figure 7a shows how the thermodynamic cycle changes under off-design operations. When the *P_eva_* decreases from 2000 kPa to 1900 kPa, the *ṁ_R245fa_* is decreased by 13.4%, which brings a significant increase of the superheating degree Δ*T* from 10.7 °C to 37.0 °C. *P_con_* in contrast is almost insusceptible with a variation of less than 1.9%. Figure 7b–d show the heat transfer profiles of the the exhaust gas, thermal oil and R245fa. When *P_eva_* decreases from 2000 kPa to 1900 kPa, the thermal oil temperature *T_a_* and *T_b_* both rise slightly while the *T_g-out_* increases from 424.0 Kto 429.4 K. This is due to the heat transfer deterioration of the evaporator caused by the decline of working fluid mass rate. In particular, when the *P_eva_* increases to 2040 kPa, the degree of superheating Δ*T* drops down to 0. If *P_eva_* increases further, the heat source is incapable of heating all the working fluid to a vapor state. From this research, it can be concluded that the degree of superheating Δ*T* is sensitive to the variation of operational variable (*P_eva_*), which can be a reliable control indicator for safety. In contrast, the condensing pressure is almost immune to the variation of evaporating pressure. It would relieve the challenge of system controller since *P_con_* is not susceptible to the variation of operational variable.

**Figure 8:**
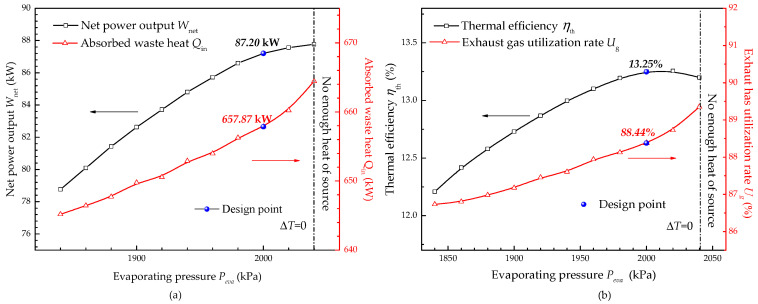
Effect of evaporating pressure on system performance: (**a**) Net power output and absorbed waste heat; (**b**) Thermal efficiency and exhaust gas utilization rate.

Figure 8 shows the variation of system performance with evaporating pressure. The absorbed waste heat *Q_in_* decreases from 657.87 kW (design point) to 645.21 kW as *P_eva_* decreases from 2000 kPa to 1840 kPa. It can be explained that the heat transfer coefficient of the working fluid side in the evaporator decreases due to the decline of working fluid mass flow rate according to Equation (30). Meanwhile, the exhaust gas utilization rate decreases from 88.44% to 86.73%. 

As for the net power output and thermal efficiency, with the rise of evaporating pressure, the net power output increases first and then becomes smooth while the thermal efficiency increases first and then decreases. It can be explained that the rise of evaporating pressure surely results in an increase of the thermal efficiency. However, when the evaporating pressure exceeds the design value, although the turbine ratio is high, the isentropic efficiency of the turbine markedly decreases, which results in a thermal efficiency drop. The net power output trend can be explained by a similar reason.

**Figure 9:**
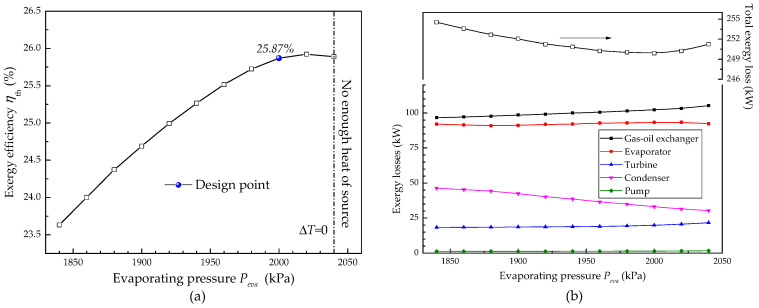
Effect of evaporating pressure on exergy efficiency and exergy losses of each component: (**a**)Exergy efficiency; (**b**) Exergy losses of each component.

Figure 9 shows the effect of evaporating pressure on exergy efficiency. Like the thermal efficiency, the exergy efficiency appears to increase first and then decrease with the rise of evaporating pressure. It is clear that with the rise of the evaporating pressure, the exergy loss of the gas-oil exchanger and the turbine increases while the exergy loss of condenser declines as shown in Figure 9b. Hence, the total exergy loss of the system appears to decrease first and then increase. The minimum exergy loss of the system can be obtained under design conditions.

### 4.2. Engine Load Effect on Energy Performance of MC/ORC System

**Figure 10:**
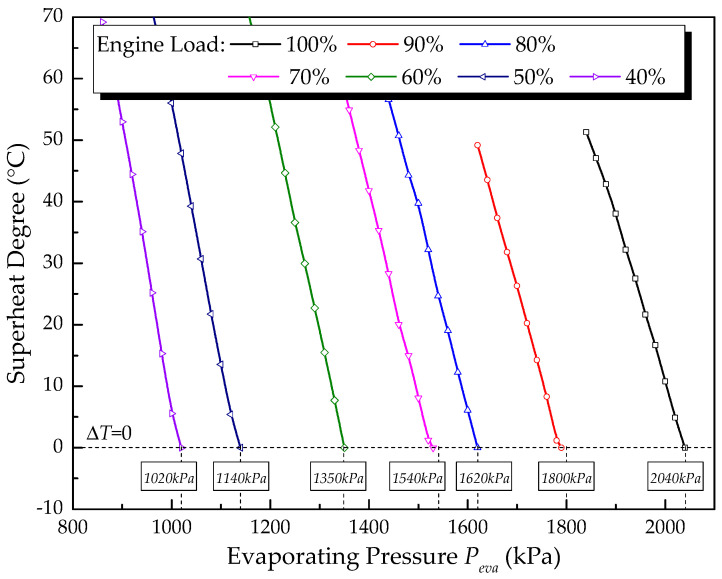
Variation of superheat degree with evaporating pressure under each engine load.

Similar calculations to those in Section 4.1 are done for all seven engine conditions in this section. The feasible operation points under each engine load are obtained and the off-design performance of MC/ORC is discussed when the engine switches from full load to partial loads. Figure 10 shows the variations of degree of superheating with evaporating pressure under different engine loads. Under all seven engine loads, the degree of superheating decreases constantly with the rise of evaporating pressure. The maximum feasible evaporating pressure under each engine load is obtained at the operation boundary limit of Δ*T* = 0. When the engine switches from 100% load to 40% load, the maximum feasible evaporating pressure decreases from 2040 kPa to 1020 kPa. This happens because the exhaust gas temperature and mass flow rate both decrease with the drop of engine load (shown in Table 2), the working fluid mass rate should be reduced to ensure that working fluid is completely vaporized at the outlet of the evaporator. Hence, the maximum feasible evaporating pressure declines with a drop of engine load. The value of the maximum feasible evaporating pressure under each engine load is noted in Figure 10.

**Figure 11:**
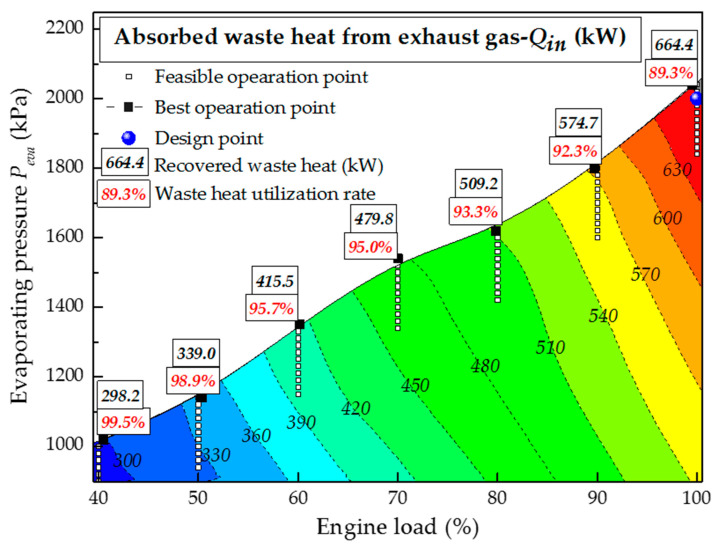
Effect of engine load on absorbed waste heat and exhaust gas utilization rate.

Figure 11 shows the effect of engine load on absorbed waste heat *Q_in_*. When the engine switches from 100% load to 40% load, the temperature and mass rate of the exhaust gas decrease, which inevitably results in a reduction of maximum *Q_in_*, from 664.4 kW to 298.2 kW. To further clarify the heat recovery capability of the MC/ORC system, the maximum *U_g_* values under different engine loads are also shown in Figure 11. It is worth noting that the maximum *U_g_* increases from 89.3% to 99.5% with the drop of engine load from 100% to 40%. This is because the heat exchanged in the evaporator when the engine operates under a partial load is significantly smaller than the design value, hence the *T_g-out_* inevitably drops below the design value. Thanks to the high exhaust outlet temperature under the design conditions, the system is free from acid corrosion of the exhaust gas. It can be concluded that the MC/ORC system can recover waste heat effectively even if the engine load decreases sharply.

**Figure 12:**
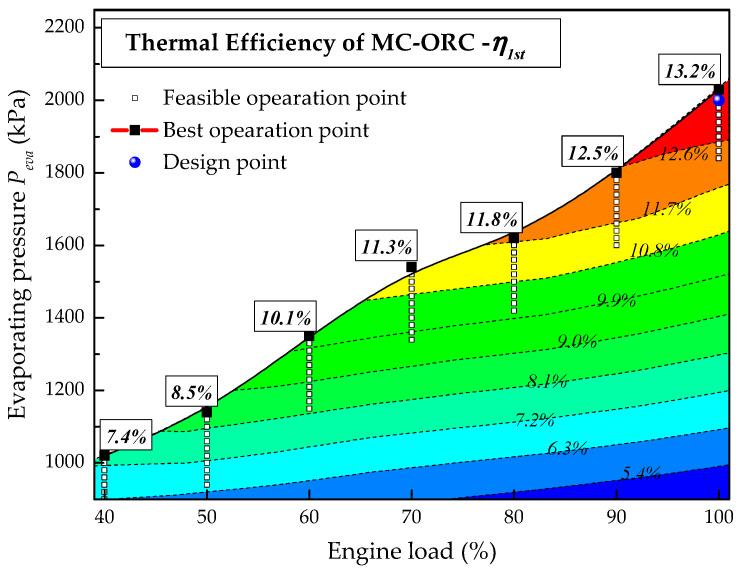
Effect of engine load on the thermal efficiency of the system.

**Figure 13:**
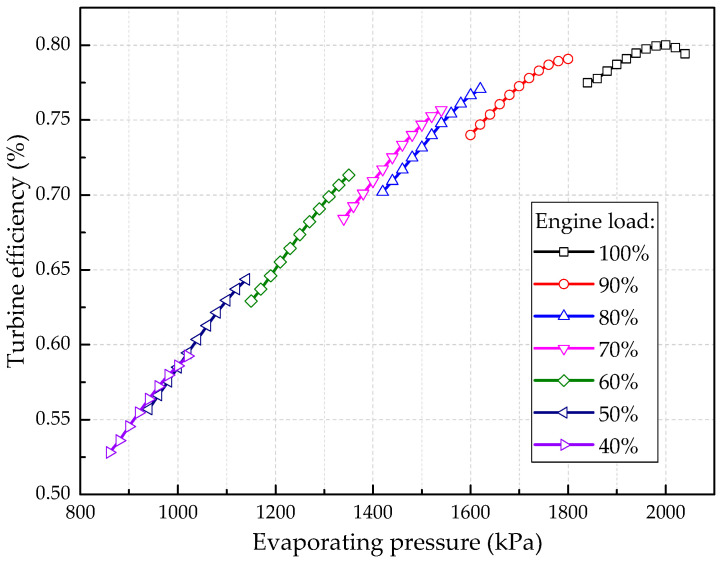
Effect of engine load on turbine efficiency under different engine loads.

Figure 12 shows the effect of engine load on the thermal efficiency of the system. Under all engine loads except 100% load, the thermal efficiency increases constantly with the rise of evaporating pressure and the maximum thermal efficiency occurs at the operation boundary limit of Δ*T* = 0. The maximum thermal efficiency under each engine load is noted in Figure 12. It is clear that the maximum thermal efficiency decreases as the engine load drops. This result can be explained as follows: although the design evaporating pressure is 2000 kPa, the feasible evaporating pressure is lower than the design value when the engine operates under partial load conditions as shown in Figure 10. The drop of evaporating pressure surely results in a decrease of the thermal efficiency. In addition, the isentropic efficiency of the turbine also drops since it deviates from the design conditions as shown in Figure 13. Under 100% engine load, the turbine efficiency increases first and then decreases with the rise of evaporating pressure and the maximum value (80%) is obtained under design conditions. When the engine switches from full load to partial load, the turbine efficiency is significantly lower than the design value due to the drop of evaporating pressure and working fluid flow rate, which also results in a drop of the thermal efficiency.

**Figure 14:**
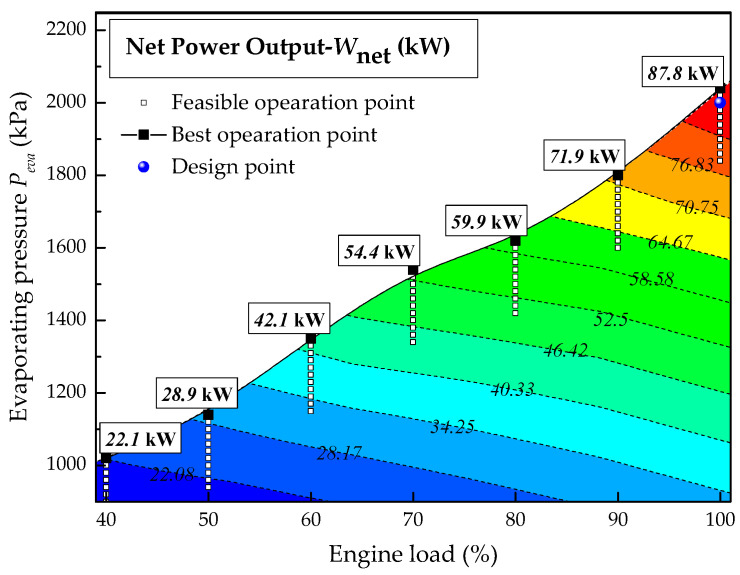
Effect of engine load on the net power output of the system.

As for the net power output shown in Figure 14, the blue point in Figure 14 represents the *W*_net_ (87.2 kW) under design conditions. The maximum net power outputs at 100% to 40% engine load are 87.8, 71.9, 59.9, 54.4, 42.1, 28.9 and 22.1 kW, respectively. The net power output under partial load of the engine is always lower than the design value. The trend of net power output is caused by the joint influence of absorbed waste heat and turbine efficiency.

### 4.3. Engine Load Effect on Exergy Performance of MC/ORC System

**Figure 15:**
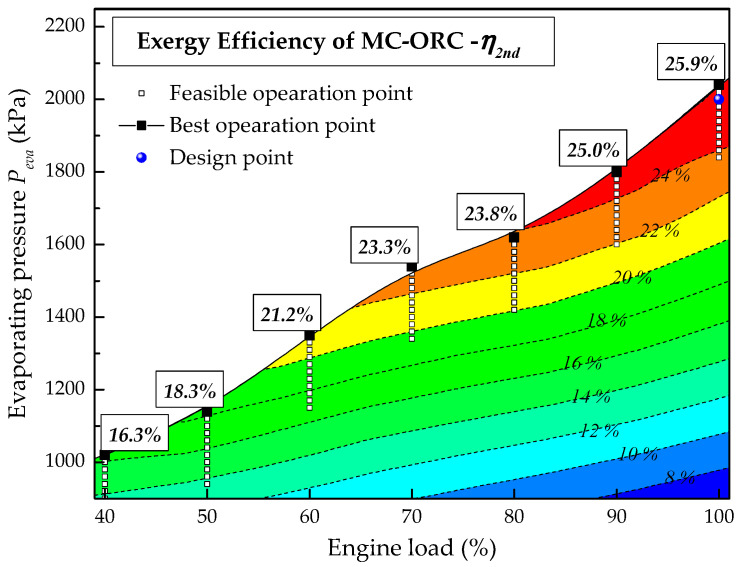
Effect of engine load on exergy efficiency of system.

Figure 15 shows the effect of the engine load on the exergy efficiency of the system. Like the thermal efficiency, under all engine loads except 100% load, the exergy efficiency increases with the rise of evaporating pressure. From 100% engine load to 40% engine load, the maximum exergy efficiencies are 25.9%, 25.0%, 23.8%, 23.3%,21.2%,18.3% and 16.3%, respectively.

**Figure 16:**
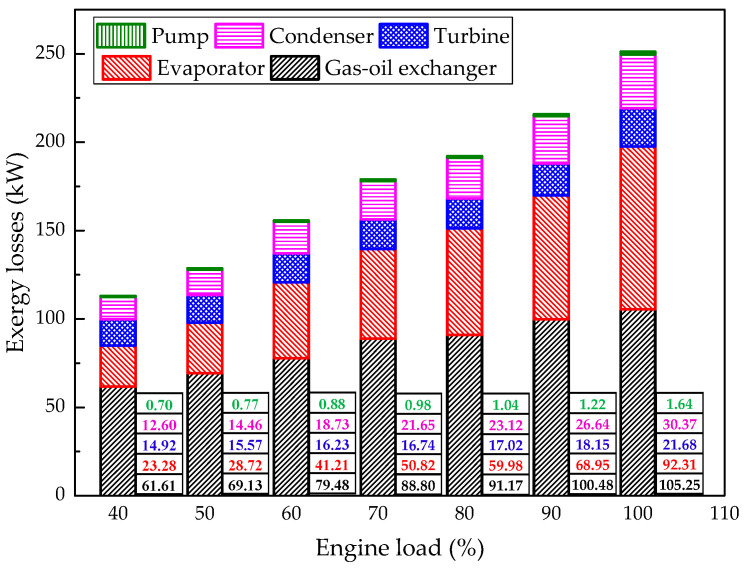
Exergy losses of each component under each engine load.

In order to get a better understanding of the irreversibility of the MC/ORC system, the exergy losses and of each component are plotted in Figure 16. Among the five components, the irreversibility in the gas-oil exchanger and evaporator are higher than those of the others. The exergy losses of the gas-oil heat exchanger and evaporator both decrease with the drop of engine load. This is due to the fact that the exergy entering the system decreases sharply with the drop of engine load. Under 60%–100% engine load, the exergy loss of the condenser is higher than that of the turbine. However, under 40%–50% engine load, the exergy loss of the turbine is larger than that of the condenser. The exergy loess of the pump is negligible.

**Figure 17:**
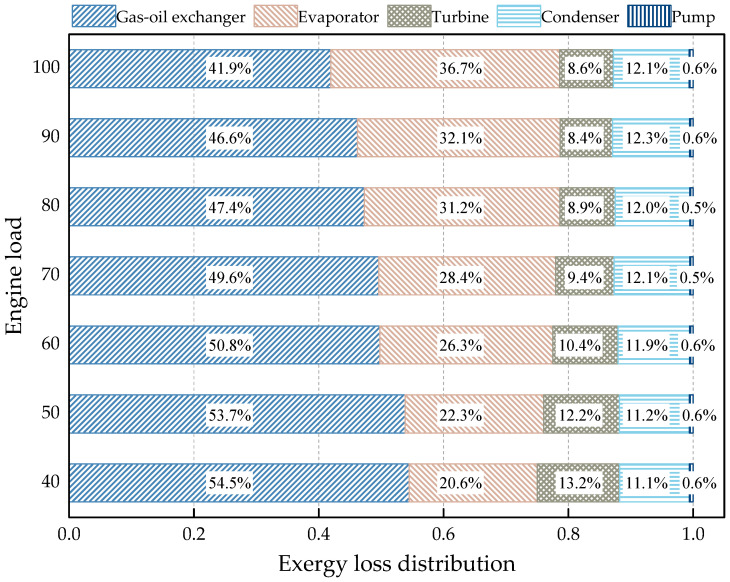
Contribution of components to the total exergy destruction under each engine load.

Figure 17 gives the relative contributions of the components to the total exergy destruction under each engine load. When the engine switches from 100% to 40% engine load, the proportion of the gas-oil heat exchanger increases from 41.9% to 54.5%, while the proportion of the evaporator decreases from 36.7% to 20.6%. This is because the evaporating pressure and working fluid mass rate both decline with the drop of engine load, which results in a decrease of the thermal-oil temperature at the evaporator inlet and outlet. Hence, the temperature difference in the evaporator decreases relatively. For the turbine, the proportion of exergy destruction increases from 8.6% to 13.2% since the drop of turbine efficiency results in severe irreversibility during the expansion process. In contrast, the proportion of the condenser declines from 12.1% to 11.1%. This is because the cooling load of the system decreases with the drop of engine load, which results in a decline of the condensing pressure. Hence, the temperature difference in the condenser also decreases. 

## 5. Conclusions

In this paper, a Medium Cycle/Organic Rankine Cycle (MC/ORC) system was designed based on a stationary CNG engine at rated load to recover exhaust gas waste heat. An off-design MC/ORC system model was presented by interconnecting the component models, which allows the prediction of the off-design behavior of the MC/ORC. The effect of operational variables and engine load on system performance is analyzed from the aspects of energy and exergy. The main conclusions that can be drawn are as follows:

1. The degree of superheating Δ*T* is sensitive to the operational variables, which can be a reliable control indicator for an actual MC/ORC system for safety. In contrast, the condensing pressure was almost immune to the variation of the operational variables. This would relieve the challenge of the system controller. This conclusion is helpful for the design of control systems in engineering practice.
2. For heat recovery capability, the exhaust utilization rate increases from 89.4% to 99.5% with the drop of engine load from 100% to 40%. The MC/ORC system can recover waste heat effectively even if the engine load decreases sharply, however, the maximum net power output, thermal efficiency and exergy efficiency decrease nearly linearly with the drop of engine load.
3. The exergy losses in the gas-oil heat exchanger and evaporator are always the main causes of exergy destruction. Considering the contribution of components to the total exergy destruction, the proportions of the gas-oil exchanger and turbine increase while the proportions of evaporator and condenser decrease with the drop of engine load.

The analytical methods used in this paper could be applied to other waste heat recovery cases with unstable heat sources.

## Figures and Tables

**Table 1:** Specifications of the 8300ZLD-1 CNG engine.

Item	Parameter	Unit
Model	8300ZLD-1	-
Ignition method	Spark plug ignition	-
Cylinder number	8	-
Displacement	215	L
Bore × stroke	300 × 380	mm
Rated speed	600	rpm
Rated power	1000	kW
Max. power	1100	kW

**Table 2:** Experiment data of the stationary CNG engine under different load.

Parameter	Value						
Speed/(r/min)	600	600	600	600	600	600	600
Engine load	40%	50%	60%	70%	80%	90%	100%
Effective power/(kW)	400	500	600	700	800	900	1000
Heat consumption rate of gas/(MJ/kWh)	13.09	11.76	11.08	10.59	10.20	10.26	9.85
Exhaust temperature/(K)	751.15	768.15	782.15	793.15	803.15	809.15	813.15
Intake air volume flow rate/(m^3^/h)	1774	2145	2465	2748	3120	3510	4180
Exhaust volume flow rate/(m^3^/h)	1911	2310	2654	2959	3380	3800	4500
Exhaust mass flow rate/(kg/s)	0.7272	0.8020	0.9752	1.1112	1.1736	1.3194	1.5625
Engine thermal efficiency/(%)	27.5	30.6	32.3	34.0	35.3	35.0	36.5

**Table 3:** The main design parameter of the MC/ORC system.

Items	Unit	Value
Environment temperature	K	298.15
Environment pressure	kPa	101.3
**Hot source**		
Temperature of exhaust gas from CNG engine	K	813.15
Mass flow rate of exhaust gas	kg/s	1.5625
Acid dew point of exhaust gas	K	373.15
**Thermal oil circuit**		
Working fluid type [32]	-	Dowtherm Q
Thermal oil temperature of evaporator inlet	K	523.15
Mass flow rate of thermal oil	kg/s	2.3
**ORC circuit**		
Working fluid type	-	R245fa
Evaporating pressure	kPa	2000
Condensation temperature	°C	35
Mass flow rate of working fluid	kg/s	2.6
Superheated degree of evaporator	K	10
Expander isentropic efficiency	-	0.8
Pump isentropic efficiency	-	0.7
Pinch point temperature difference of evaporator	K	30
Pinch point temperature difference of condenser	K	10
**Cold sink**		
Mass flow rate of cooling water	kg/s	23.0
Cooling water temperature of condenser inlet	°C	25

**Table 4:** Heat exchangers design data.

Exchanger Data	Unit	Gas-Oil Exchanger	Evaporator	Condenser
Types	-	BEM	BEM	BEM
Tube inside fluid	-	Thermal-oil	R245fa	Water
Tube outside fluid	-	Exhaust gas	Thermal-oil	R245fa
Baffle type	-	Single segmental	Single segmental	Single segmental
Tube pattern	°	60	60	60
Tube-side passes	*Nt*	2	2	2
Shell-side passes	*Ns*	1	1	1
Tube outside diameter	*d_o_*/mm	12	14	16
Tube thickness	*th*/mm	2	2	1
Pitch between tubes	*st*/mm	30	20	20
Number of tube	*n_t_*	800	240	120
Tube length	mm	1230	1180	1870
Shell diameter	*D_shell_*/mm	921	342	245
Baffle space	mm	460	171	122
Baffle cut	mm	230	85	61

**Table 5:** Turbomachine design data.

Parameter	Pump	Turbine	
Fluid	R245fa	R245fa	
Design mass flow rate *ṁ_des_*/kg/s	-	2.6	
Design volume flow rate ${\dot{V}}_{des}$/m^3^/h	7.16	-	
Stodola’s coefficient *K_s_*	-	0.0056	
Design isentropic efficiency	0.7	0.8	
Coefficients	*a* = −0.439	*a*_1_ = −1.519	*a*_2_ = 0.001
	*b* = 0.466	*b*_1_ = 0.027	*b*_2_ = −0.776
	*c* = 0.453	*c*_1_ = 2.123	*c*_2_ = 1.574
	*d* = 0.519	*d*_1_ = 0.219	*d*_2_ = 0.203

## Nomenclature

CCHP	Combined cool heat and power
CNG	Combined cool heat and power
DES	Distributed energy system
ICE	Internal combustion engine
NG	Natural gas
MC/ORC	Medium Cycle/organic rankine cycle
UR	Utilization ratio
*U*	Heat transfer coefficient (Kw/m^2^ K)
*A*	Area (m^2^)
*c_p_*	Constant pressure specific heat (kJ/kg·K)
*d*	Diameter (m)
*E*	Exergy (W)
*f*	Friction factor
*g*	Gravity acceleration (m/s^2)^
*G*	Mass flux of the working fluid (kg/m^2^ s)
*h*	Enthalpy (KJ/kg)
*h*	Heat transfer coefficient (W/(m^2^·K))
*Ks*	Stodola’s coefficient
*L*,*l*	Length (m)
*ṁ*	Mass flow rate (kg/s)
*P*	Pressure (kPa)
*Pr*	Prandtl number
*Q*	Heat flux (kW)
*r*	Latent heat (kJ/kg)
*Re*	Reynolds number
*s*	Specific entropy (kJ·kg^−1^·K^−1^)
*T*	Temperature (K)
Δ*T*	Superheat degree (K)
*v*	Velocity (m/s)
$\dot{V}$	Volumetric flow rate (m^3^/h)
*W*	Output work (kJ)
*x*	Steam quality
*λ*	Thermal conductivity (W/(m·K))
*η*	Efficiency
*ρ*	Density (kg/m^3^)
*μ*	Dynamic viscosity
*con*	Condenser
*cw*	Cooling water
*des*	Design value
*g*	Exhaust gas
*eva*	Evaporator
*l*	Liquid
*shell*	Shell side
*tube*	Tube side
*t*	Turbine
*i*	Inside
*in*	Inlet
*is*	Isentropic
*o*	Outside
*oil*	Thermal oil
*out*	Outlet
*pp*	Pump
*v*	Vapor
*wf*	Working fluid
